# Deficiency in IL-33/ST2 Axis Reshapes Mitochondrial Metabolism in Lipopolysaccharide-Stimulated Macrophages

**DOI:** 10.3389/fimmu.2019.00127

**Published:** 2019-02-01

**Authors:** Huadan Xu, Liankun Sun, Yichun He, Xiaofeng Yuan, Junqi Niu, Jing Su, Dong Li

**Affiliations:** ^1^Key Laboratory of Pathobiology, Ministry of Education, Department of Pathophysiology, College of Basic Medical Sciences, Jilin University, Changchun, China; ^2^Department of Neurosurgery, China-Japan Union Hospital, Jilin University, Changchun, China; ^3^Department of Pediatrics, Affiliated Hospital of Changchun University of Chinese Medicine, Changchun, China; ^4^Department of Hepatology, The First Hospital of Jilin University, Changchun, China; ^5^Department of Immunology, College of Basic Medical Sciences, Jilin University, Changchun, China

**Keywords:** IL-33, ST2, macrophage, PGC1α, ATP

## Abstract

The polarization and function of macrophages play essential roles in controlling immune responses. Interleukin (IL)-33 is a member of the IL-1 family that has been shown to influence macrophage activation and polarization, but the underlying mechanisms are not fully understood. Mitochondrial metabolism has been reported to be a central player in shaping macrophage polarization; previous studies have shown that both aerobic glycolysis and oxidative phosphorylation uniquely regulate the functions of M1 and M2 macrophages. Whether IL-33 polarizes macrophages by reshaping mitochondrial metabolism requires further investigation. In this work, we examined the mitochondrial metabolism of bone marrow-derived macrophages (BMDMs) from either wild type (WT), *Il33*-overexpressing, or IL-33 receptor knockout (*St2*^−/−^) mice challenged with lipopolysaccharide (LPS). We found that after LPS stimulation, compared with WT BMDMs, *St2*^−/−^ BMDMs had reduced cytokine production and increased numbers and activity of mitochondria *via* the metabolism regulator peroxisome proliferator-activated receptor-C coactivator-1 α (PGC1α). This was demonstrated by increased mitochondrial DNA copy number, mitochondria counts, mitochondria fission- and fusion-related gene expression, oxygen consumption rates, and ATP production, and decreased glucose uptake, lactate production, and extracellular acidification rates. For *Il33*-overexpressing BMDMs, the metabolic reprogramming upon LPS stimulation was similar to WT BMDMs, and was accompanied by increased M1 macrophage activity. Our findings suggested that the pleiotropic IL-33/ST2 pathway may influence the polarization and function of macrophages by regulating mitochondrial metabolism.

## Introduction

Macrophages play important role in every stage of immune responses, in both healthy and disease settings. Macrophages can be polarized to different phenotypes according to their surrounding microenvironment, and each phenotype has its own properties and unique functions. Generally, macrophages are cataloged into two major phenotypes based on their glucose metabolism and functions: lipopolysaccharide (LPS)—or IFNγ-stimulated inflammatory M1 type macrophages, which convert arginine into nitric oxide by inducible nitric oxide synthase (iNOS); and Interleukin (IL)-4-stimulated anti-inflammatory and pro-resolution M2 type macrophages, which convert arginine to ornithine by arginase-1 (1, 2). In addition to these differences in arginine metabolism, different subsets of macrophages have distinguishable mitochondrial activities.

It has been reported that mitochondrial metabolism is a central player in shaping macrophage polarization. Previous reports have shown that glycolysis is reprogrammed in LPS-stimulated M1 type macrophages due to impaired mitochondrial function, leading to a Warburg-like effect (aerobic glycolysis), which can be swiftly activated (3). M1 type macrophages not only use glucose to generate ATP, but also use the energy and metabolites (e.g., pyruvate) generated from glycolysis to fuel the pentose phosphate pathway (PPP) and fatty acid acetyl coenzyme A (acetyl-CoA) synthesis, eventually resulting the stabilization of hypoxia inducible factor 1α (HIF1α) and the production of pro-inflammatory cytokines (4). Conversely, IL-4-stimulated M2 type macrophages are supported by mitochondrial oxidative phosphorylation (OXPHOS) (2). As the different glucose metabolism pathways determine macrophage functions, reshaping them might alter these functions and even change immune responses from detrimental to beneficial or *vice versa* (5).

IL-33, which belongs to the IL-1 cytokine family and bind to the receptor ST2, was discovered in 2005 and has been extensively researched since (6). Because IL-33 is a pleiotropic cytokine, it can activate or polarize many types of immune cells, promoting either pro-inflammatory or anti-inflammatory immune responses depending on the specific microenvironment. The interaction of IL-33 and macrophages has been reported to be essential for all stages of immune responses, including the initiation (7), lasting (8–10), and final resolution stages (11, 12).

IL-33 can contribute to macrophage polarization in both pro-M1 and pro-M2 settings (13). Although the underlying mechanisms are not fully understood, IL-33 may polarize macrophages through its canonical ST2/MYD88/IRAK1/4 pathway, or potentially through the binding of full-length IL-33 with transcription factors that alter macrophage phenotypes. Our group previously found that the IL-33/ST2 pathway influenced macrophages proliferation and activity (Li et al. 11 and unpublished data), both of which are known to be closely associated with mitochondrial metabolism. We also found that peroxisome proliferator-activated receptor-coactivator 1α (PGC1α) played a key role in altering mitochondrial metabolism *via* promoting mitochondrial biogenesis (14). Thus, whether IL-33/ST2 signaling can sufficiently alter mitochondrial metabolism to change macrophage functions is worth investigating.

In this study, we used bone marrow-derived macrophages (BMDMs) from wild-type (WT), *St2*^−/−^, and *Il33*-overexpressing mice, and we stimulated these macrophages with LPS to investigate the role of the IL-33/ST2 pathway in mitochondrial metabolism and macrophage function. We found that the IL-33/ST2 pathway was required for the LPS-induced metabolic reprogramming of macrophages. These results might provide further insight into how macrophages initiated proper responses after encountering stimuli.

## Materials and Methods

### Mice

Specific pathogen-free 6–9-week-old male BALB/c mice were purchased from Beijing Vital River Laboratory Animal Technology Co., Ltd. (Beijing, China) and housed in specific pathogen-free conditions at Jilin University (15). *St2*^−/−^ mice were kindly provided by Prof. Weihua Xiao from the University of Science and Technology of China (Hefei, China), and *Il33* transgenic mice were kindly provided by Prof. Ying Sun from Capital Medical University (Beijing, China). Both strains were in the BALB/c background (11). All animal experiments were performed in accordance with the National Guidelines for Experimental Animal Welfare and with approval of the Animal Welfare and Research Ethics Committee at Jilin University (Changchun, China).

### Cell Culture

Primary BMDMs were generated as previously described (11). Briefly, murine bone marrow cells were harvested and cultured in RPMI 1640 supplemented with 10% fetal calf serum, 2 mM L-glutamine, 100 U/ml penicillin, 100 μg/ml streptomycin, 0.05 M 2-ME, and 10 ng/ml macrophage colony-stimulating factor (M-CSF; PeproTech, Rocky Hill, NJ, US) for 6 d in a humidified cell culture incubator containing 5% CO_2_ at 37°C. All tissue culture reagents and lipopolysaccharide (LPS, L6529) were purchased from Sigma-Aldrich (St. Louis, MO, USA) unless otherwise stated.

### Quantitative Real-Time PCR (qPCR)

Total RNA was extracted from cultured BMDMs using TRIzol Reagent (Thermo Fisher Scientific, Waltham, MA, US). Genomic DNA digestion and reverse transcription were performed using the EasyScript First-Strand cDNA Synthesis SuperMix (TransGen Biotech, Beijing, China) according to the manufacturer's instructions. For qPCR analyses, cDNA were amplified using a TransStart Green qPCR SuperMix (TransGen Biotech). The cycling parameters were 94°C for 5 s, 50°C−60°C for 15 s and 72°C for 10 s for 40 cycles. A melting-curve analysis was then performed to check PCR specificity. CT values were measured during the exponential amplification phase. Relative expression levels (defined as fold change) of target genes were determined using the 2–ΔΔCT method. *Actb* was used as an internal control. Expression levels were normalized to the fold change detected in the corresponding control cells, which was defined as 1.0. The primers used were as follows: *Il1a* forward 5′-ACG GCT GAG TTT CAG TGA GAC C-3′ and reverse 5′-CAC TCT GGT AGG TGT AAG GTG C-3′; *Il1b* forward 5′-TGG ACC TTC CAG GAT GAG GAC A-3′ and reverse 5′-GTT CAT CTC GGA GCC TGT AGT G-3′; *Nos2* forward 5′-GCC TCG CTC TGG AAA GA-3′ and reverse 5′-TCC ATG CAG ACA ACC TT-3′; *Ifng* forward 5′-CAG CAA CAG GCA AGG CGA AAA AGG-3′ and reverse 5′-TTT CCG CTT CCT GAG GCT GGA T-3′. *Mfn1* forward 5′-CCT ACT GCT CCT TCT AAC CCA-3′ and reverse 5′-AGG GAC GCC AAT CCT GTG A-3′; *Mfn2* forward 5′-GTG GGC TGG AGA CTC ATC G-3′ and reverse 5′-CTC ACT GGC GTA TTC CGC AA-3′; *Opa1* forward 5′-ACA GCA AAT TCA AGA GCA CGA-3′ and reverse 5′-TTG CGC TTC TGT TGG GCA T-3′; *Dnm1l* forward 5′-ACC GGG AAT GAC CAA AGT ACC-3′ and reverse 5′-TGG GAT TAC TGA TGA ACC GAA GA-3′; and *Fis1* forward 5′- AGA GCA CGC AAT TTG AAT ATG CC-3′ and reverse 5′-ATA GTC CCG CTG TTC CTC TTT-3′.

### Relative Mitochondrial Copy Number

Mitochondrial copy numbers were measured as previously described (14). Briefly, BMDMs were cultured on coverslips for 24 h, and then treated with LPS for 72 h. Relative mitochondrial DNA (mtDNA) copy number was measured by qPCR on total DNA extracted using the TIANamp Genomic DNA Kit (Tiangen, Beijing, China). Primer sequences for the mitochondrial segment were: *mt-Nd1* forward 5′-CAC CCA AGA ACA GGG TTT GT-3′ and reverse 5′-TGG CCA TGG GAT TGT TGT TAA-3′. Primer sequences for the single-copy nuclear control were: *18S* forward 5′-TAG AGG GAC AAG TGG CGT TC-3′ and reverse 5′-CGC TGA GCC AGT CAG TGT-3′. Mitochondrial copy number was calculated relative to nuclear DNA using the following equations:

(1) $$\begin{array}{r} \Delta \text{CT}=\text{Mitochondrial CT}-\text{NuclearCT} \end{array}$$

(2) $$\begin{array}{r} \text{Relativemitochondrial DNA content}=2^{-\text{ΔCT}} \end{array}$$

### Determining Glucose Uptake and Lactate Production

BMDMs cells were treated with LPS (0, 0.1, 0.5, and 1.0 μg/ml) for 72 h, and then the culture medium was collected for glucose and lactate measurements with glucose and lactate assay kits (Beyotime, Haimen, Jiangsu, China), respectively. Data were normalized to the corresponding total protein amounts from each sample, as previously described (16).

### Oxygen Consumption Rate (OCR) and Extracellular Acidification Rate (ECAR) Analysis

A total of 8 × 10^4^ BMDMs were seeded into 96-well plates and incubated overnight to allow adherence. The following day, different concentrations of LPS were added into the indicated wells for 24 h. Each treatment was repeated in three wells. OCR and ECAR were measured using oxygen-sensitive (Mito-Xpress) and pH-sensitive (pH-Xtra) fluorescent probes (Luxcel Bioscience, Cork, Ireland) as previously described (16).

### Determining Intracellular ATP Production

Intracellular ATP production was measured using the Enhanced ATP Test Kit (Beyotime). Briefly, BMDMs were treated with LPS (0, 0.1, 0.5, and 1.0 μg/ml) for 72 h, and then cells were collected and the assay was performed according to the manufacturer's instructions. Data were normalized to the corresponding total protein amounts from each sample, as previously described (17).

### Measuring Mitochondrial Membrane Potential

Mitochondrial membrane potential (MMP) in BMDM was determined using a JC-1 probe contained within the Mitochondrial Membrane Potential Assay Kit (Beyotime). At 6 h post-LPS treatment, cells were incubated with 1 ml of 1 × JC-1 for 30 min at 37°C in the dark, and the ratio of cells positive for red fluorescence (JC-1 polymer-positive, indicating intact MMP) to those positive for green fluorescence (monomeric JC-1, indicating loss of MMP) was determined by flow cytometry using a BD Accuri C6 (BD Biosciences, Franklin Lakes, NJ, US) and then analyzed with FlowJo software (Version 10.0.7; FlowJo, LLC, OR, US) as previously described (18).

### Mitochondrial Imaging by Confocal Microscopy

BMDMs were cultured on coverslips for 24 h, and then treated with LPS (0, 0.1, 0.5, and 1.0 μg/ml) for 72 h. The fluorescent dye MitoTracker RED (Thermo Fisher Scientific) was used to monitor mitochondrial content in living cells according to the manufacturer's instructions. Then cells were imaged with an Olympus FV 1000 laser-scanning confocal microscope (Olympus, Tokyo, Japan).

### Cytokine Measurements

The concentrations of cytokines in cell culture media were determined using ELISA kits (Thermo Fisher Scientific) according to manufacturers' instructions.

### Western Blotting Analysis

BMDMs were treated with LPS (0, 0.1, 0.5, and 1.0 μg/ml) for 24 h or 48 h, then the cells were lysed in RIPA buffer (Thermo Scientific) containing protease inhibitors (Roche, Basel, Switzerland). Protein concentrations were estimated by the BCA protein assay (Thermo Scientific). Proteins were then incubated at 70°C for 10 min in reducing SDS sample buffer and 30 μg of protein lysate per lane was run through NuPAGE® Novex® 4–12% Bis-Tris Protein Gels (Thermo Scientific) and transferred to Hybond ECL membranes (GE Healthcare, Chicago, IL, US). Membranes were blocked for 1 h in 5% non-fat dried milk in double distilled PBS (DPBS) and incubated overnight with the appropriate primary antibody at 4°C. Membranes were then washed in DPBS/Tween 20 (Bio-Rad Laboratories, Hercules, CA, US) and incubated with the appropriate secondary antibody. Detection was performed by ECL Western Blotting Detection Reagents (Bio-Rad Laboratories). Antibody against PGC1, DRP1, MFN1, MFN2, OPA1, and FIS1 were obtained from Santa Cruz Biotechnology (Dallas, TX, US); β actin and all secondary antibodies were obtained from Proteintech (Wuhan, Hubei, China).

### Statistical Analysis

Data are expressed as means ± standard error (SEM). Statistical significance between two groups was analyzed by One-way ANOVA followed by Student's *t*-test using Prism software (GraphPad Software, La Jolla, CA, US). N.S. represents no statistical difference between the compared groups; ^*^ represents *P* < 0.05 and was considered statistically significant. All experiments were repeated at least three times.

## Results

### *ST2* Deficiency Impaired Macrophage Responses Upon LPS Stimulation With Less Glucose Uptake and Lactic Acid Generation

**Figure 1:**
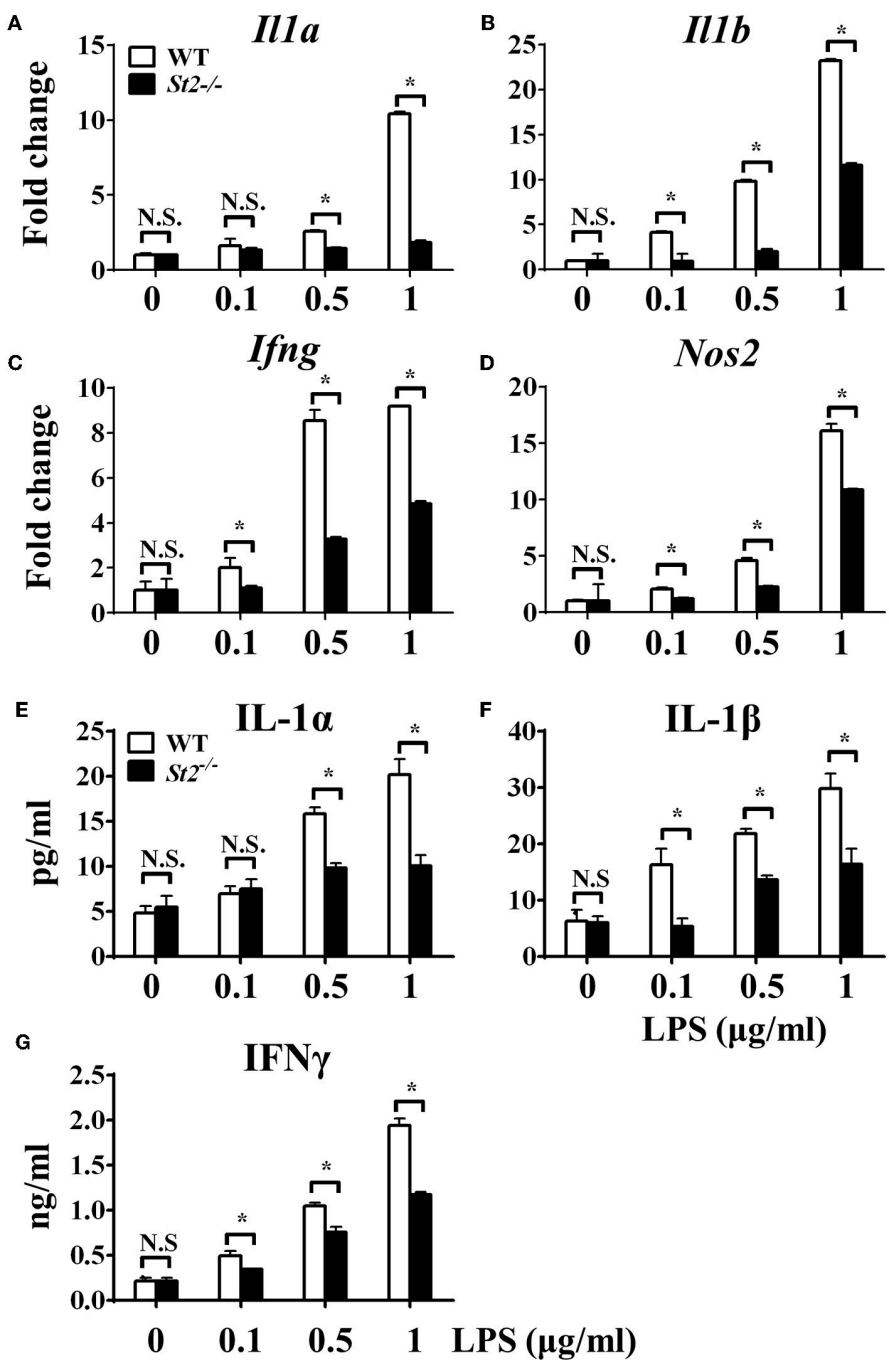
ST2 deficiency impaired macrophages responses upon LPS stimulation. BMDMs from BALB/c or *St2*^−/−^ mice were stimulated with LPS (0, 0.1, 0.5, and 1.0 μg/ml) for 72 h. Total RNA was isolated and the expression of *Il1a* **(A)**, *Il1b* **(B)**, *Ifng* **(C)**, and *Nos2* **(D)** was evaluated by qPCR; the concentration of IL-1α **(E)**, IL-1β **(F)**, and IFNγ **(G)** in the supernatant was evaluated by ELISA. Vertical bars = SEMs (*n* = 3 per group per experiment). N.S., no significant difference; ^*^p < 0.05.

**Figure 2:**
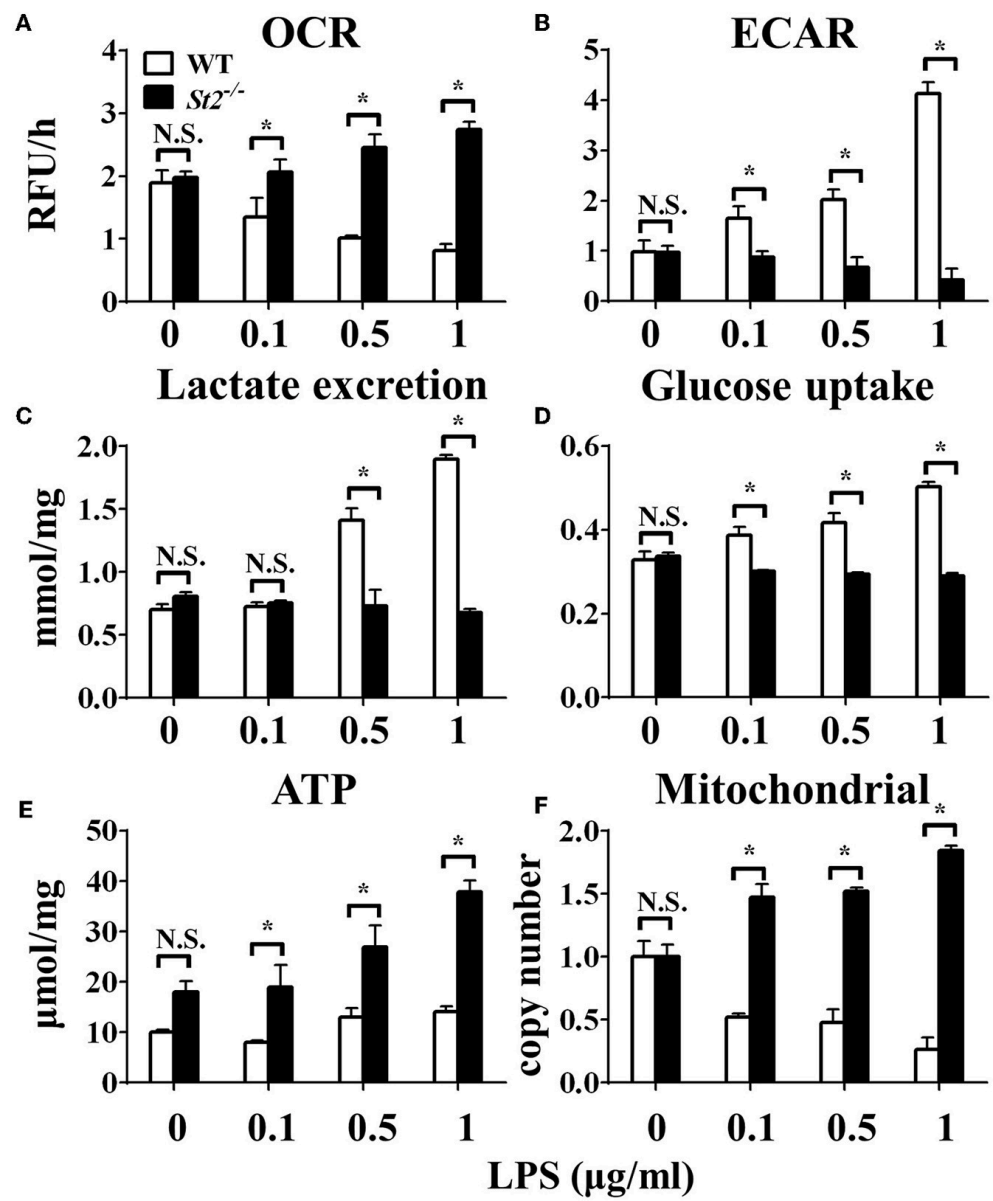
ST2 deficiency affected the metabolism of macrophages upon LPS stimulation. BMDMs from BALB/c or *St2*^−/−^ mice were stimulated with LPS (0, 0.1, 0.5, and 1.0 μg/ml) for 24 h and the **(A)** extracellular oxygen consumption rate (OCR) was immediately measured by fluorescence intensity of a typical oxygen probe; **(B)** extracellular acidification rate (ECAR) was measured by incubation at 37°C for 3 h. The lactate excretion **(C)**, glucose uptake **(D)**, ATP **(E)**, and relative mitochondrial DNA copy numbers **(F)** of BMDMs were measured after 72 h stimulation of LPS (0, 0.1, 0.5, 1.0 μg/ml). Vertical bars = SEMs (*n* = 3 per group per experiment). N.S., no significant difference; ^*^p < 0.05.

To investigate the role of IL-33/ST2 signaling in LPS-stimulated macrophages, BMDMs were exposed to different LPS doses, and metabolic characteristics and cytokine production were monitored. As reported before, *St2*-deficient BMDMs were not as responsive as WT BMDMs, as demonstrated by decreased *Il1a, Il1b, Nos2* and *Ifng* expression as measured by qPCR (Figures 1A–D) and the concentration of IL-1α, IL-1β, IFNγ in supernatant by ELISA (Figures 1E–G). *St2* deficiency increased the OCR (Figure 2A) of BMDMs, while reducing their ECAR (Figure 2B), lactate acid generation (Figure 2C), and glucose consumption (Figure 2D). These results indicated that macrophages undergo aerobic glycolysis (a Warburg-like effect) after they have been active by LPS; however, in the absence of IL-33/ST2 signaling, macrophages increase OXPHOS after LPS stimulation. Subsequent experiments showed that this Warburg-like effect was not induced by mitochondrial damage (Supplementary Figure 1).

### ST2 Deficiency Was Associated With Enhanced Mitochondrial Function

**Figure 3:**
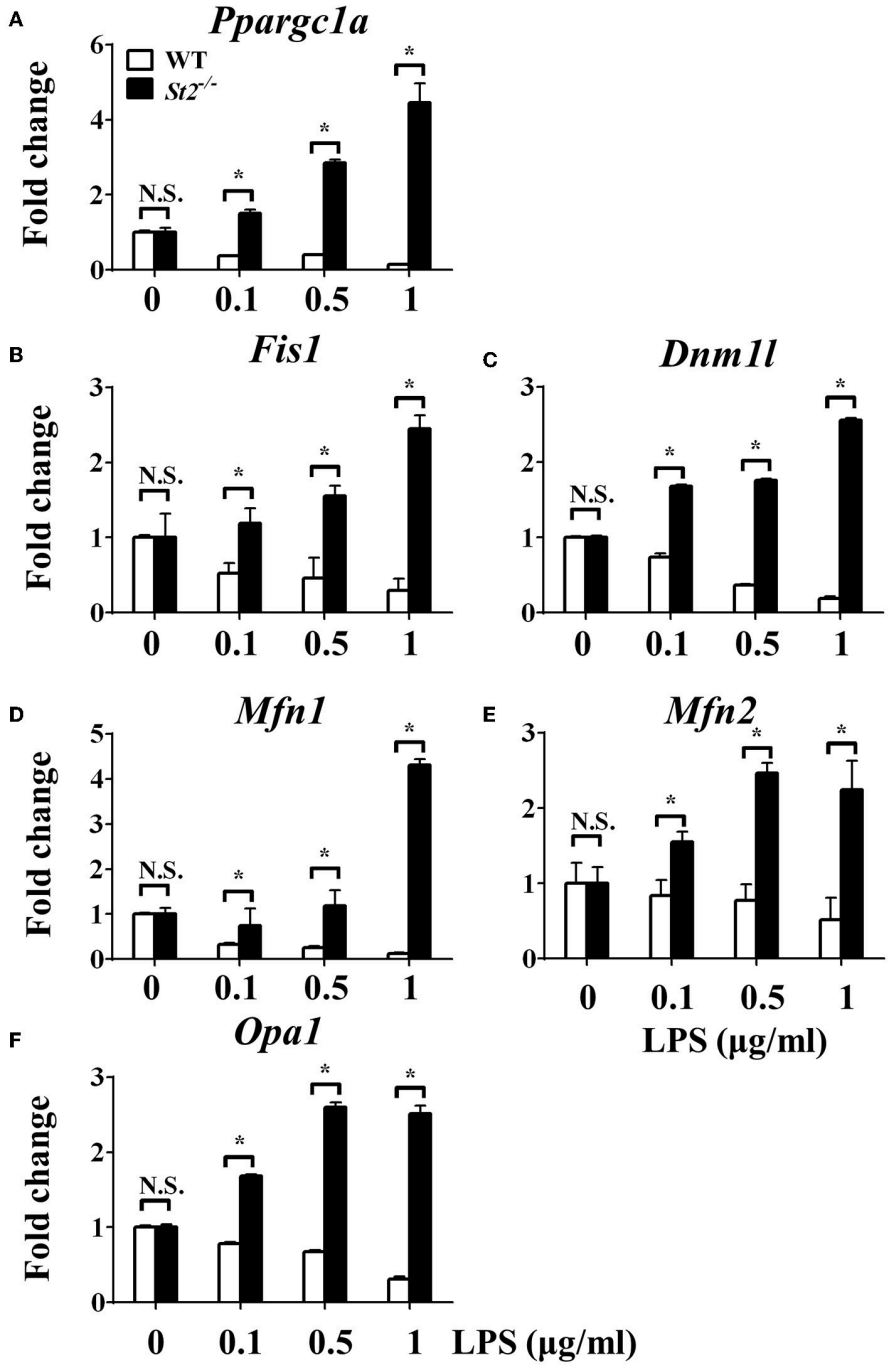
ST2 deficiency was associated with enhanced mitochondrial biogenesis. BMDMs from BALB/c or *St2*^−/−^ mice were stimulated with LPS (0, 0.1, 0.5, and 1.0 μg/ml). After 72 h, total RNA was isolated and the expression of *Ppargc1a* **(A)**, *Fis1* **(B)**, *Dnm1l* **(C)**, *Mfn1* **(D)**, *Mfn2* **(E)**, and Opa1 **(F)** were evaluated by qPCR, respectively. Vertical bars = SEMs (*n* = 3 per group per experiment). N.S., no significant difference; ^*^p < 0.05.

**Figure 4:**
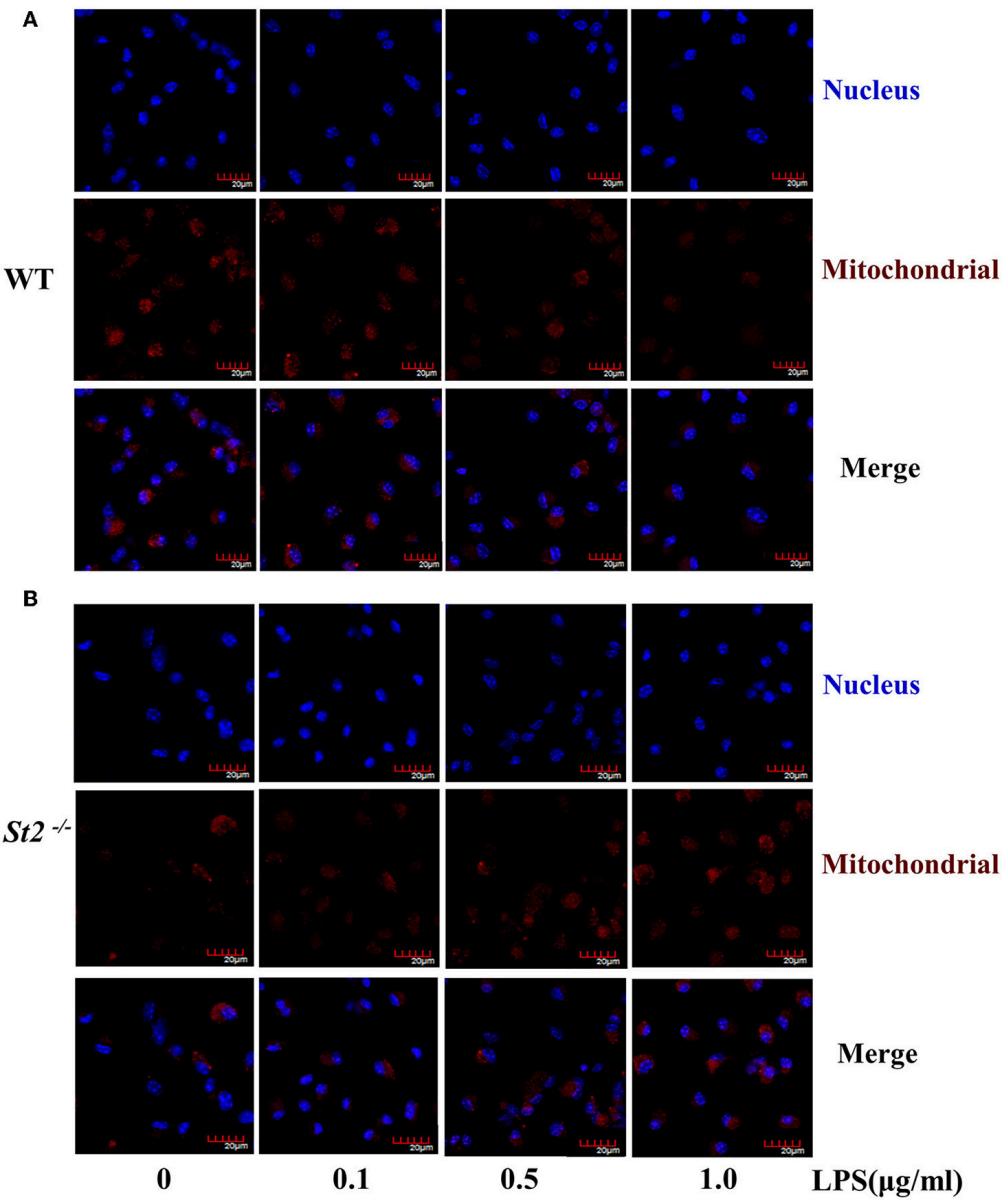
ST2 deficiency was associated with increased mitochondria mass. BMDMs from WT **(A)** or *St2*^−/−^ **(B)** mice stimulated with LPS (0, 0.1, 0.5, and 1.0 μg/ml) for 72 h. Mitochondrial mass was observed with confocal laser microscopy in BMDMs stained with MitoTracker Red (scale bar: 20 μm). Data are representative of three experiments.

To investigate the mechanism underlying the metabolic reprogramming of macrophages that lacked IL-33/ST2 signaling, we evaluated mitochondrial activity. Both the number and activity of mitochondria were increased in *St2*-deficient BMDMs, as shown by more mitochondrial gene copies and ATP production in *St2*^−/−^ BMDMs compared with WT BMDMs (Figures 2E,F). The expression of *Ppargc1a*, which encodes PGC-1α, a master regulator of mitochondrial biogenesis, was also measured; the results showed that LPS increased PGC-1α expression only in *St2*^−/−^ BMDMs (Figure 3A). Next, the induction of mitochondrial fission- and fusion-associated genes (*Fis1, Dnm1l* and *Mfn1, Mfn2, Opa1*, respectively) was determined by qPCR (Figures 3B–F) and western blotting (Supplementary Figure 2). LPS only enhanced the expression of these genes in *St2*^−/−^ BMDMs but not in WT. These changes in mitochondria were also confirmed by fluorescent staining. Mitochondrial numbers were reduced by LPS in WT but increased in *St2*^−/−^ BMDMs (Figure 4).

### Overexpressing IL-33 Promoted Macrophage Responses Upon LPS Stimulation With More Glucose Uptake and Lactic Acid Generation

**Figure 5:**
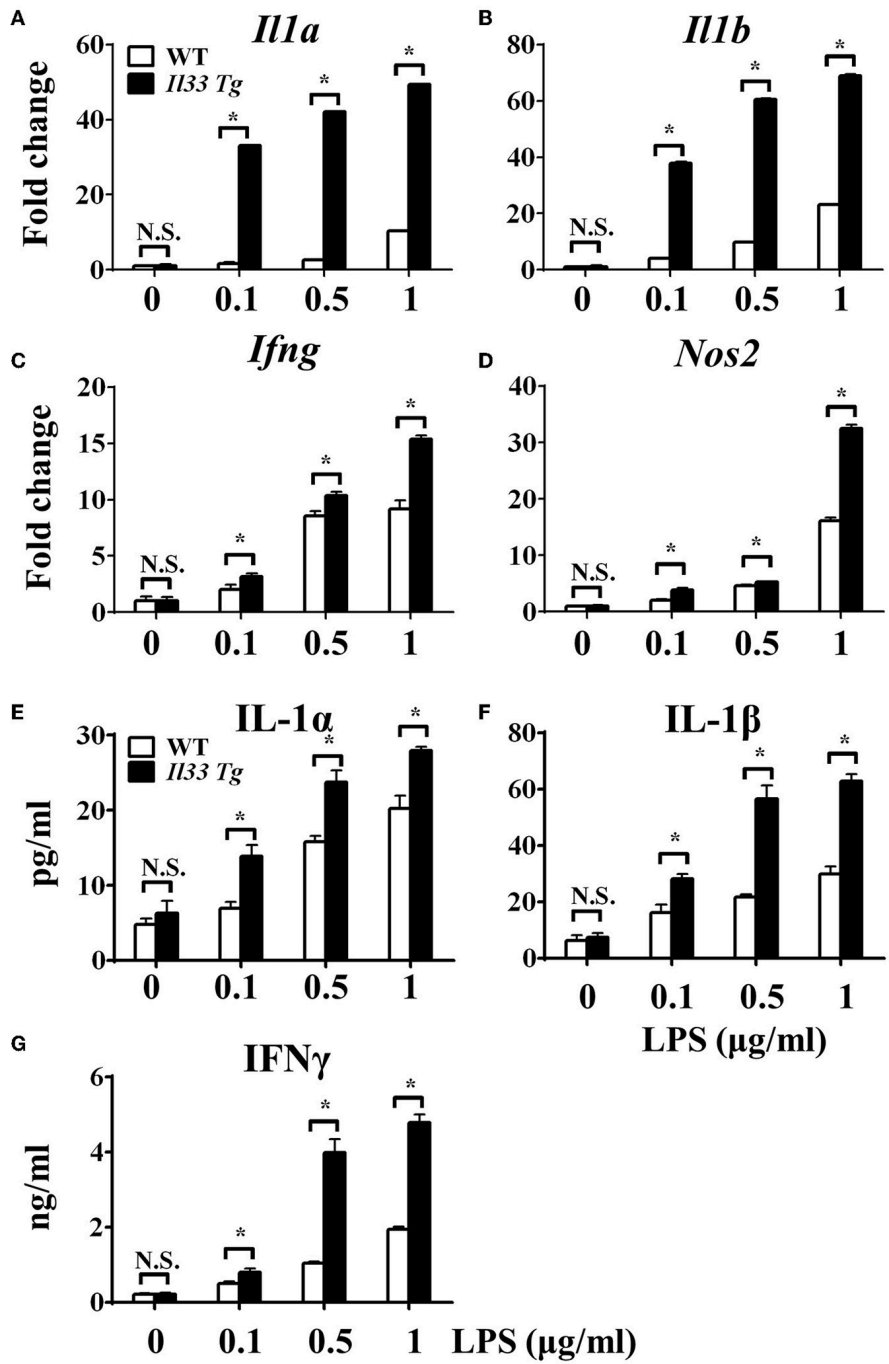
IL-33 over-expression promoted the macrophages responses upon LPS stimulation. BMDMs from BALB/c or *Il33* over-expressing (*Il33 Tg*) mice were stimulated with LPS (0, 0.1, 0.5, and 1.0 μg/ml) for 72 h. Total RNA was isolated and the expression of *Il1a* **(A)**, *Il1b* **(B)**, *Ifng* **(C)**, and *Nos2* **(D)** was evaluated by qPCR; the concentration of IL-1α **(E)**, IL-1β **(F)**, and IFNγ **(G)** in the supernatant was evaluated by ELISA. Vertical bars = SEMs (*n* = 3 per group per experiment). N.S., no significant difference; ^*^p < 0.05.

**Figure 6:**
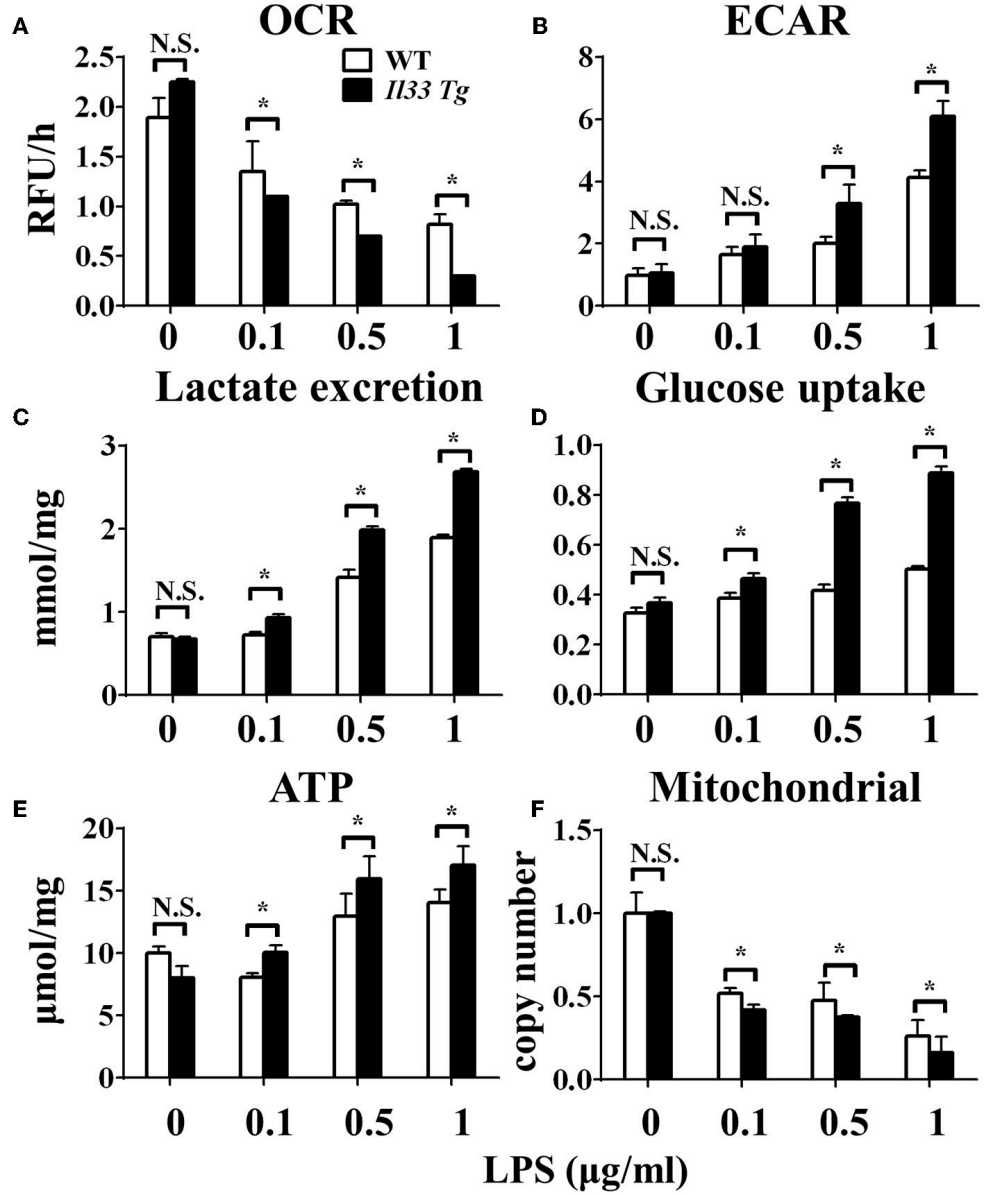
IL-33 over-expression affected the metabolism of macrophages upon LPS stimulation. BMDMs from BALB/c or *Il33* over-expressing (*Il33 Tg*) mice were stimulated with LPS (0, 0.1, 0.5, and 1.0 μg/ml) for 24 h and the **(A)** extracellular oxygen consumption rate (OCR) was immediately measured by fluorescence intensity of a typical oxygen probe; **(B)** extracellular acidification rate (ECAR) was measured by incubation at 37°C for 3 h. The lactate excretion **(C)**, glucose uptake **(D)**, ATP **(E)** and relative mitochondrial DNA copy numbers **(F)** of BMDMs were measured after 72 h stimulation of LPS (0, 0.1, 0.5, 1.0 μg/ml). Vertical bars = SEMs (*n* = 3 per group per experiment). N.S., no significant difference; ^*^p < 0.05.

After we established that LPS induced OXPHOS in macrophages in the absence of IL-33/ST2 signaling by increasing the proliferation, fission, and fusion of mitochondria, possibly due to the induction of PGC-1α, we next determined whether IL-33 overexpression could alter these effects. After both WT and *Il33*-overexpressing BMDMs were stimulated with different doses of LPS, IL-1α, IL-1β, iNOS, and IFNγ production were measured and metabolic changes in the cells were monitored. IL-33 overexpression enhanced the production of pro-inflammatory cytokines and iNOS in LPS-stimulated macrophages, as measured by qPCR and ELISA (Figures 5A–G). Furthermore, IL-33 overexpression reduced the OCR of macrophages and increased the ECAR (Figures 6A,B), lactate acid production (Figure 6C), and glucose uptake (Figure 6D). These results indicated that, in contrast to ST2 deficiency, IL-33 overexpression was associated with enhanced macrophage function by enhancing the Warburg-like effects that were triggered by LPS.

### IL-33 Overexpression Was Associated With Reduced Mitochondrial Fission and Fusion

**Figure 7:**
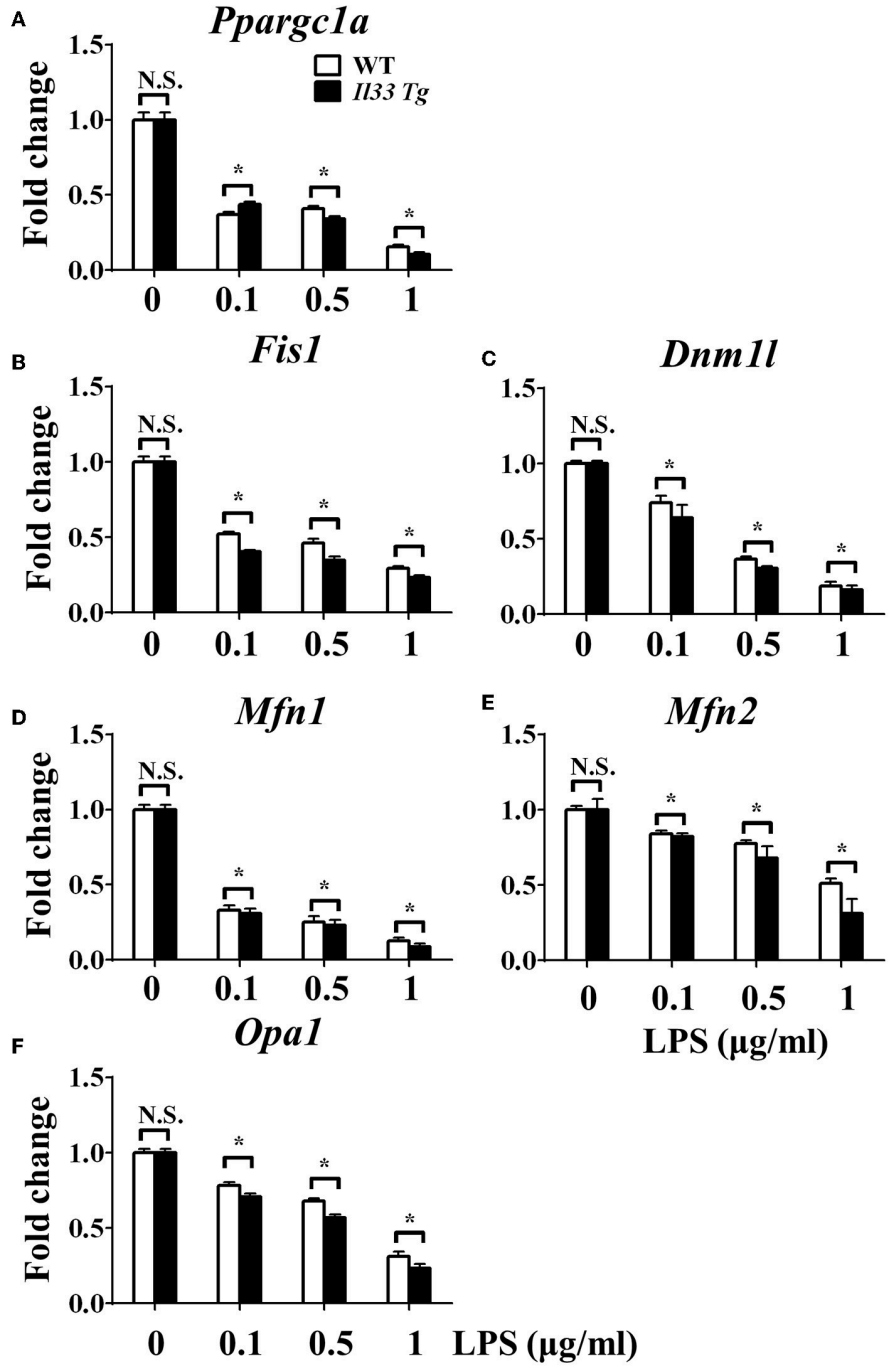
IL-33 over-expression was associated with reduced mitochondrial biogenesis. BMDMs from BALB/c or *Il33* over-expressing (*Il33 Tg*) mice were stimulated with LPS (0, 0.1, 0.5, and 1.0 μg/ml). After 72 h, total RNA was isolated and the expression of *Ppargc1a* **(A)**, *Fis1* **(B)**, *Dnm1l* **(C)**, *Mfn1* **(D)**, *Mfn2* **(E)**, and Opa1 **(F)** was evaluated by qPCR, respectively. Vertical bars = SEMs (*n* = 3 per group per experiment). N.S., no significant difference; ^*^p < 0.05.

We further investigated whether IL-33 overexpression changed the metabolism of LPS-stimulated macrophages through a similar mechanism as ST2 deficiency. IL-33 overexpression was associated with fewer mitochondrial gene copies (Figure 6F), less fission (Figures 7B,C) and fusion (Figures 7D–F; Supplementary Figure 2), but was still associated with higher ATP production (Figure 6E). The changes in PGC-1α expression in LPS-stimulated *Il33*-overexpressing BMDMs were similar to WT BMDMs (Figure 7A). These results indicated that IL-33 enhanced the metabolic changes in macrophages following LPS stimulation *via* decreasing mitochondrial proliferation, fission, and fusion.

## Discussion

The metabolic changes in macrophages upon contacting different stimuli are essential for macrophage polarization and function in both physiological and pathological conditions (4). The IL-33/ST2 pathway is known to direct macrophages toward different phenotypes when combined with different stimuli *via* previously unknown mechanisms (19, 20). Here, we showed for the first time that the IL-33/ST2 pathway may directly reshape central carbon metabolism in macrophages.

IL-33 plays pleiotropic role in human immunopathology (21, 22). For example, it can be beneficial for sepsis (7, 12), malaria (23), obesity related inflammation (24), autoimmune-related uveitis (25), and experimental autoimmune encephalomyelitis (10). In these settings, IL-33 had protective effects by inducing neutrophils, type 2 innate lymphoid cells, regulatory T cells and the production of IL-17 and IFNγ, depending on the specific settings. Conversely, IL-33 is a detrimental factor in other settings, such during autoantibody-induced arthritis (26–29), eosinophilic asthma (8, 9), cancer (30, 31), early-stage colitis (32), and lung fibrosis (11). IL-33 might exacerbate these diseases through the induction of eosinophils, type 2 innate lymphoid cells, mast cells, the production of pro-inflammatory and pro-fibrotic cytokines, inducing mucositis, or directly promoting the proliferation and metastasis of cancer cells. These examples highlight the multitude of roles and underlying mechanisms downstream of IL-33/ST2 signaling in both healthy and disease settings. Further studies designed at understanding these mechanisms are required and could possibly provide insight into how to manipulate the immune system to treat these diseases.

One of the possible reasons for the controversial roles of the IL-33/ST2 pathway in inflammation could be explained by interactions between IL-33/ST2 signaling and different macrophages. It is well established that macrophages have distinguished functions in shaping immune responses (33), and previous reports have shown that IL-33 can polarize macrophages to the pro-inflammatory M1-like subset or the anti-inflammatory and pro-resolution M2-like subset. Furthermore, blocking IL-33/ST2 signaling inhibited macrophage responses after LPS stimulation (34, 35), while exogenous IL-33 enhanced the M1-like polarization of LPS-stimulated macrophages (35). These results are similar to what we showed in this work (Figures 1, 4). Furthermore, macrophages generate IL-33 in response to LPS stimulation (36, 37), and exogenous IL-33 enhances the polarization of macrophages to a M2-like phenotype when combined with other type 2 cytokines (8). These studies prove the close relationship between IL-33/ST2 signaling and macrophage activation and polarization.

It has been reported that the activation and polarization of macrophages requires metabolic reprogramming (2). IL-33 has been shown to upregulate hypoxia- HIF-1α (29), which in-turn modulates glucose metabolism and macrophage function. IL-33 can also signal in an autocrine manner, which can create a positive-feedback loop for the IL-33/ST2 pathway (38). But the underlying mechanisms remained undiscovered, which might be due to the different nuclear functions of full length IL-33 compared with the mature cytokine form of IL-33 (39, 40). Our groups' previously work proved that PGC1α produced metabolic changes in cells *via* promoting mitochondrial proliferation and activity (14), which were also closely related to macrophage responses to LPS stimulation. WT macrophages could down-regulate PGC1α to limit mitochondrial proliferation, which promotes glycolysis over OXPHOS. Aerobic glycolysis or the Warburg effect is less efficient at ATP production compared with OXPHOS, but glycolysis generates several metabolites that are useful for protein synthesis and the reactive oxygen species generated by NADPH oxidase (41). When PGC1α is upregulated, mitochondrial proliferation is promoted, and cells use OXPHOS as the primary method of generating ATPs. OXPHOS is so efficient at ATP generation that might deplete substrates for other important biosynthetic reactions inside the cell.

In this work, we investigated the metabolic reprogramming of LPS-stimulated macrophages in the absence or excess of IL-33/ST2 signaling. We found that the IL-33/ST2 pathway played an important role in the metabolic switch, from OXPHOS to glycolysis (Warburg effect), in LPS-stimulated macrophages by altering PGC1α levels. We also determined that this metabolic reprogramming did not result from mitochondrial damage, as MMP was not significantly changed by LPS stimulation or *St2* knockout. These results could provide further insight into the interactions between IL-33/ST2 and macrophages, and might help in future pharmaceutical approaches to treat immune dysfunctions.

## Author Contributions

JS and DL contributed to experimental design, securing funds, and manuscript preparation. LS and JN contributed to the supervision of the study and manuscript preparation. HX, YH, and XY contributed experimentation and data analysis.

### Conflict of Interest Statement

The authors declare that the research was conducted in the absence of any commercial or financial relationships that could be construed as a potential conflict of interest.

## Supplementary Material

Supplementary Figure 1 ST2 deficiency reduced the mitochondrial membrane potential of LPS stimulated macrophages. BMDMs from BALB/c **(A)** or *St2*^−/−^ **(B)** mice were stimulated with LPS (0, 0.1, 0.5, and 1.0 μg/ml) for 6 h. Mitochondrial membrane potential staining with JC-1 was detected by flow cytometry. Upper panel showed representative histogram figures and lower panel showed representative dot plots. Data are representative of three experiments.

Supplementary Figure 2 IL-33/ST2 pathway affected the fusion and fission of mitochondria in LPS stimulated macrophages. BMDMs from BALB/c, *St2*^−/−^ or *Il33* over expression (*Il33 Tg*) mice were stimulated with LPS (0 and 1.0 μg/ml) for 24 or 48 h. The expression of mitochondrial fission and fusion-related proteins (PGC1, DRP1, MFN2, MFN1, OPA1, and FIS1) were analyzed by western blot. Data are representative of three experiments.
