# Supplementary figures and images for: Deficiency in IL-33/ST2 Axis Reshapes Mitochondrial Metabolism in Lipopolysaccharide-Stimulated Macrophages
Source: Front Immunol. 2019 Feb 1;10:127. doi: 10.3389/fimmu.2019.00127 (PMC6367255; doi:10.3389/fimmu.2019.00127)

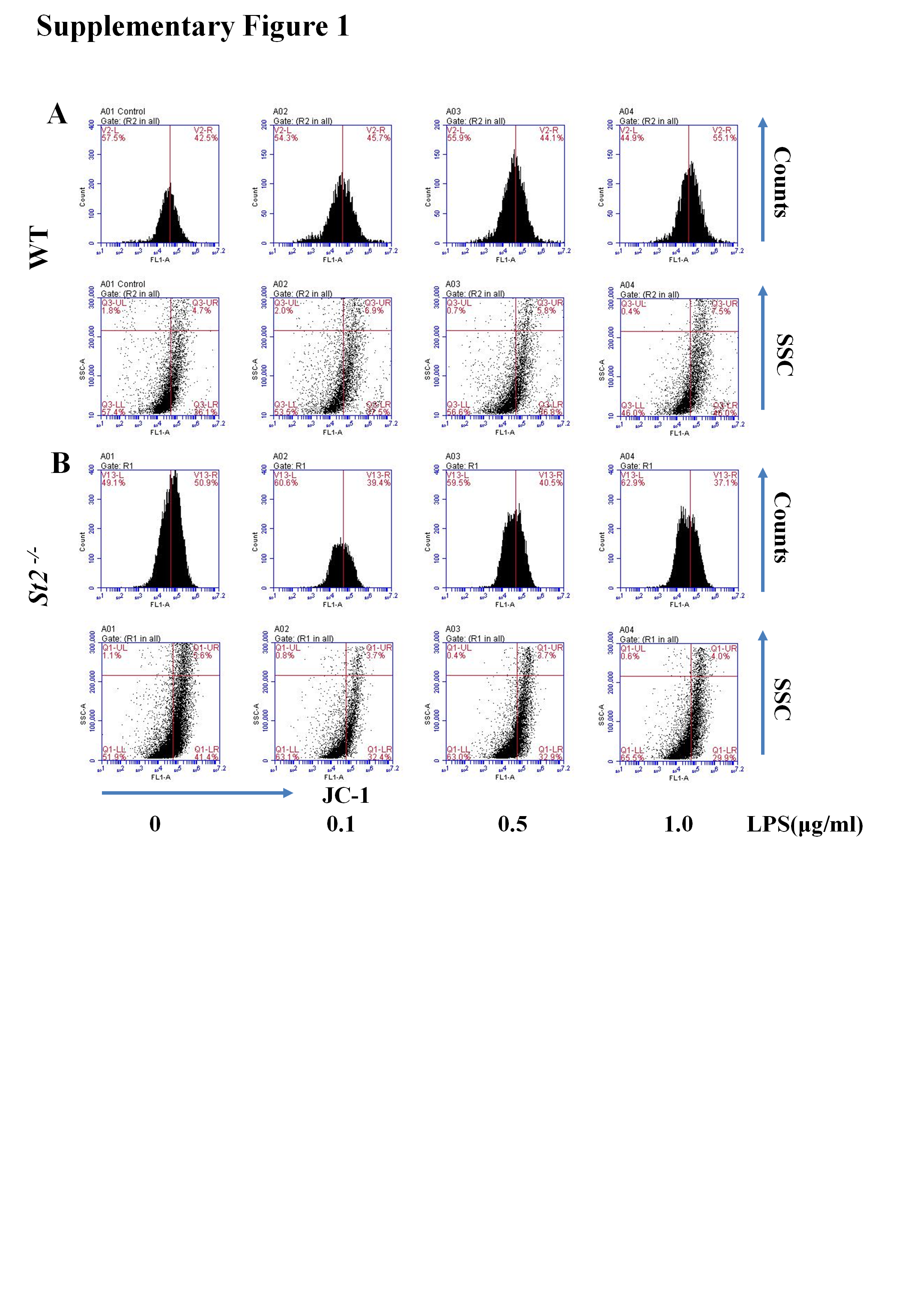

Supplement: Supplementary Figure 1 — ST2 deficiency reduced the mitochondrial membrane potential of LPS stimulated macrophages. BMDMs from BALB/c (A) or St2−/− (B) mice were stimulated with LPS (0, 0.1, 0.5, and 1.0 μg/ml) for 6 h. Mitochondrial membrane potential staining with JC-1 was detected by flow cytometry. Upper panel showed representative histogram figures and lower panel showed representative dot plots. Data are representative of three experiments. [file Image_1.TIF]

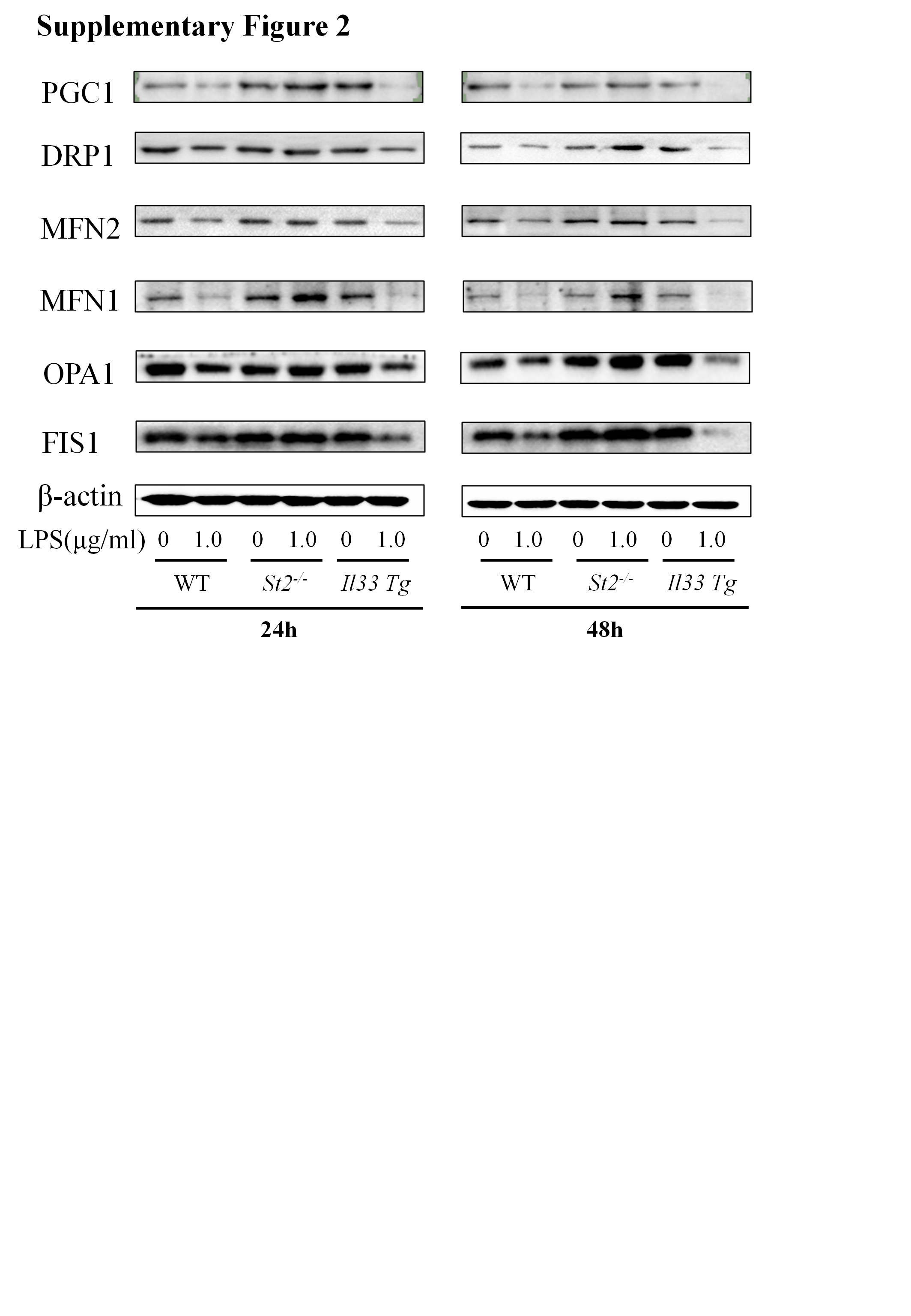

Supplement: Supplementary Figure 2 — IL-33/ST2 pathway affected the fusion and fission of mitochondria in LPS stimulated macrophages. BMDMs from BALB/c, St2−/− or Il33 over expression (Il33 Tg) mice were stimulated with LPS (0 and 1.0 μg/ml) for 24 or 48 h. The expression of mitochondrial fission and fusion-related proteins (PGC1, DRP1, MFN2, MFN1, OPA1, and FIS1) were analyzed by western blot. Data are representative of three experiments. [file Image_2.TIF]
